# When does family size matter? Sibship size, socioeconomic status and education in England

**DOI:** 10.1017/ehs.2020.54

**Published:** 2020-10-19

**Authors:** Paula Sheppard, Christiaan Monden

**Affiliations:** 1 Institute of Social and Cultural Anthropology, University of Oxford, Oxford, UK; 2 Department of Sociology, University of Oxford, Oxford, UK

**Keywords:** Parental investment, resource dilution, siblings, family size, birth order

## Abstract

There is still no clear understanding of the relationship between sibship size and child outcomes. Research from across disciplines, and across settings, reports conflicting results suggesting that the relationship is complex and ecologically dependent. Evolutionary models predict that parents will make reproductive decisions based on their ability to invest in each child, but that this is not necessarily equal across children. Here we use data from the Next Steps study linked to National Pupil Database to examine the relationship between sibship size and Key Stage 4 (GCSE) maths and English grades in England for children born in 1989/1990. We were interested to further examine if and how associations might differ at the ends of the socioeconomic spectrum and we also tested if direct measures of parental investment could mitigate any negative impact of larger families. Multilevel ordinary least squares regression models with a random effect for school show that sibship sizes are associated with school grades, as is socioeconomic status. Moreover, the association between sibship size and grades holds true across the socioeconomic spectrum. Birth order was only weakly associated with school results, and only significant in some models. Parental investment is important, however, and might offset the some of the negative impact of larger families, for both maths and English attainment.

## Graphical abstract

**Figure:**
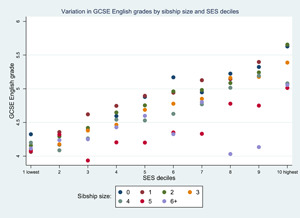

**Media summary**: Large sibships are negatively associated with GCSE grades in England, and this is seen across the socioeconomic spectrum.

## Introduction

Research from across the social sciences suggests that large sibships are associated with a number of poorer child socioeconomic outcomes such as educational attainment and adult social class (Blake, 1986, 1992; Downey, 1995; Steelman et al., 2002), and is consistently negative across contexts (Choi et al., 2020). The main argument proposed to explain this association is that parental resources are limited and are thus more thinly spread among children in large sibships. Much of this research has been carried out in the US, which questions the broader generalizability of these findings. Previous US-focused research might not be relevant in other settings such as in Europe where public spending on children is much higher (Park, 2008; Thévenon, 2011). Scrutiny of the smaller literature from Europe reveals much less consistent results. For instance, in Finland, Black et al. (2005) found that negative correlations between sibship size and education diminish greatly once they applied an instrumental variable approach (using twin births). They found rather that higher birth order far outweighed the impact of family size. Similar associations were found for children born in the 1960s and 1970s in Norway, where birth order predicted intellectual ability and educational attainment, using within-family fixed effects models to control for sibship size (Kristensen & Bjerkedal, 2010). In the same study, but comparing between families, they also showed that large sibship size and birth order were independently associated with poorer education. In a recent study using census data from England and Wales, Chan et al. (2019) report an ‘uncertain’ causal association between children of different sibship sizes and the likelihood of obtaining a university degree. Their observational (probit) models indicated a negative correlation but the causal (IV probit) model estimates became non-significant (although still in a negative direction). On the other hand, also in the UK, Bradley and Taylor (2004) report a significant association between larger sibships and poorer school attainment for both boys and girls net of a large number of family characteristics such as parental education, social class, ethnicity and neighbourhood deprivation. These studies suggest one of two things – that the relationship between sibship size and educational outcomes is (a) not causal or (b) highly context dependent.

We suggest a more in-depth approach to identify the conditions under which family size might impact upon children. We argue that exploring different contextual factors is likely to reveal hidden patterns of associations between sibship size and child education. Following an ecological approach, we argue that (a) differences between socioeconomic groups and (b) varying levels of direct parental investment will help to explain the current mixed results and shed light on the conditions under which sibship size correlations might occur. Specifically, we will assess the impact of sibship size on children in England's high school grades in English and maths. We will investigate how these relationships differ according to socioeconomic status, and also examine if, and how, direct parental investment might offset any negative effects of sibship size across socioeconomic strata.

## Theoretical framework

We take a multidisciplinary approach to thinking about the different ways and contexts that sibship size might have consequences for child education. Broadly, one can opt for producing more children and investing in them less, or conversely, producing fewer children in whom the resources can be divided more generously. This quantity–quality trade-off is known across disciplines as the resource dilution hypothesis.

The resource dilution hypothesis argues that, given limited parental resources children from larger families will, all else equal, each receive less. This argument has a number of caveats to be considered. First, unless all siblings are born at the same time, for some types of resources such as one-to-one time with a child, or food, there is an economy of scale: when an only child gains one sibling the child's resources are halved, but with the addition of a fifth sibling each existing child's resources are reduced by just 5%. The negative effect of sibship size on resource access, therefore, is expected to diminish as sibship size increases. Resource dilution patterns also probably depend on sibling age and sex configuration. Son preference, inheritance patterns and gender inequality in some cultures predict higher investment in sons (Kalmijn & Werfhorst, 2016), and in some cases, daughters (Bereczkei & Dunbar, 1997). And in families where the siblings are spaced further apart, older siblings might contribute to, rather than compete for, family resources (Kramer, 2010).

Evolutionary models of parental investment take an optimality approach, where parental reproductive decisions aim to maximize fitness within ecological, including economic, constraints. Life history theory predicts that, given limited resources, parents trade off higher investment in fewer children for more children with decreased investment in each. In contrast to the resource dilution hypothesis, here the (expected) parental access to resources is taken into account in the reproductive decision-making process. In effect, this means that sibship sizes should not be associated with child outcomes owing to differences in available resources. The cost of childrearing is considered before the children are produced. Along these lines, Mace (1998) developed a formal optimality model showing that, as the cost of children increased, parents reliably produced fewer children. Specifically, this model showed how increasing inheritance left to sons among Kenyan Gabbra pastoralists can lead to very small family sizes (providing an adaptive explanation of the demographic transition).

An ecological approach also acknowledges that limited parental resources have to be divided among children but it further considers the contextual factors. Gibbs et al. (2016) make the point that, in settings where there are generous state welfare systems, parents can offset the cost of raising children and this weakens the relationship between sibship size and child outcomes. Along these lines, Park (2008) found that the relationship between sibship size and reading literacy was much weaker in social democratic countries which prioritize child and education policies. Clearly macro-level conditions matter but differences in economic power at the family level are also expected to produce different strategies. Adaptive reasoning posits that parents with limited access to resources will balance trade-offs differently compared with those in higher-status families. In a skills-based wage-labour economy, parents who are able to invest in their children's education reap the long-term rewards of the adult child's ability to improve her own socioeconomic outlook and invest in her, and eventually her children's, status. Conversely, ‘quantity’ outcompetes ‘quality’ when the payoff to investment in education is low and short-term goals return higher rewards. High-risk contexts are therefore expected to diminish the strength of sibling competition (Desai, 1995); parents who have less control over their economic environment gain less from directing high investment in fewer children (Kaplan, 1996; Kaplan et al., 1998; Lawson & Mace, 2011).

## Background and literature

### Socioeconomic status

The relationship between family resources and family size runs in both directions. Parental resources diminish as families grow larger (Downey, 1995; Lawson & Mace, 2010). Yet lower-status individuals also produce larger families, suggesting that the quality–quantity trade-off operates a priori. This is not a simple linear relationship however; research in post-demographic transition countries shows a positive relationship between wealth and fertility *within* socioeconomic strata, even though there is a general negative trend at higher macro levels (Stulp & Barrett, 2016), although not everywhere (Jalovaara et al., 2019). We might therefore also observe variation in the association between sibship size and educational outcomes between as well as within socioeconomic groups. Specifically, we expect that the outer limits of the socioeconomic distribution will behave differently to the middle where associations might be more linear. This is because, for parents in the middle of the socioeconomic spectrum, limited resources equally distributed among siblings mean that family size should matter: children from larger families are expected to do worse at school. Parents with very low access to resources might not be able to influence their children's educational success in any substantial way and so the number of siblings one has might not make a difference. Higher-status parents, on the other hand, might be able to invest abundantly in all children and so sibship size here also becomes irrelevant. For instance, a UK study showed that fathers from higher socioeconomic status (SES) bands were more involved with their children than were lower-SES fathers (Nettle, 2008).

As far as we are aware, there have been no explicit tests of this in the UK, although some research is suggestive of heterogeneity in associations across the socioeconomic gradient. Chan et al. (2019) examined differences in the relationship between sibship size and the likelihood of the child obtaining a university degree conditional on parental education (holding a university degree). Parental university education is a good predictor of child university education (Ermisch & Francesconi, 2012), so we might not expect to see much variation when using this particular indicator of SES. And indeed, they found that parental education was the strongest predictor of child education in their study. Here we derive a composite measure of family SES (based on parental education, household income and parental occupation), which we propose will shed more light on disparities within socioeconomic groups. As well as expecting socioeconomic variation in how sibship size might impact on education, we also expect that parents will be differentially inclined to compensate for resource availability by increasing their direct input, particularly in non-material ways, across socioeconomic groups.

### Parental investment

Regardless of the socioeconomic situation families find themselves in, parents are able to support their children's education in non-material ways. Parents can invest by creating a nurturing home environment, by investing time and energy in transporting children, reading to them, supervising their homework, etc. and by taking a personal interest in their children's progress at school. Some parents may also invest materially, for instance by paying for private schooling, extra tuition in school subjects or extra-curricular activities. The amount and quality of direct parental investment is also likely to vary by family size and status. The Norwegian study cited earlier (Kristensen & Bjerkedal, 2010) was also conducted on a subsample of children whose birth order was not the same as their sibship position (social rank) in cases where an earlier-born sibling had died in infancy. This provided the opportunity to examine if birth order effects on education were due to actual birth order or social rank, and indeed they found that the child's family social rank was more important than the order they were born in. This suggests that social family dynamics, and potentially parental investment, are better explanations for child outcomes than are biological (e.g. foetal development) factors.

Evidence from the anthropological literature points to complex context-dependent effects of sibship size and birth order. Gibson and Sear (2010) showed that wealth was unevenly distributed among children from two high-fertility populations in Africa. Wealthier families invested more in earlier-born children's education while families that were less able to contribute to child education discriminated less between them. However the relationship (for wealthier families) was not linear as younger brothers were also advantaged compared with middle children. In Tanzania too, the child's household age-ranked position was more important than biological birth order or household sibship sizes for predicting school attendance, although this was biased towards younger girls, possibly because older girls could contribute to the household labour more efficiently. The opposite was true for boys where the younger boys stayed home from school (Hedges et al., 2019). Conversely, in a resource-stressed rural farming community in South Africa, Liddell et al. (2003) report that parents invested heavily in all their children's education, regardless of gender, family size or birth order. While these studies reveal that parents make investment decisions based on various socioeconomic and ecological factors which also take into account the payoffs to child education, they tell us little about *how* parents invest.

There are not many studies that look in depth at parental investment directly; rather they are inferred by child outcomes. One study in the UK has done this where Lawson and Mace (2009) derived parental scores of child-caring activities and examined how these differed across two measures of SES. They found that high- and low-income families suffered little cost (measured as how highly the parent scored on the caring index) to increasing family size, although families in the middle income bracket did. When they repeated their analysis conditional on parental education, they found similar results. Other work from the US found that the relationship between large sibship sizes and school grades was attenuated by the inclusion of measures of parental involvement and economic resources (Downey, 1995). Downey's study shows that the association between sibship size and school grades varied conditional on variables denoting parental involvement with the child (e.g. talking with the child, and knowing her friends), but not with parental material investment (such as having a computer in the home, or spending money on cultural activities), net of parental SES. Furthermore, he reports that the value of these characteristics decreases as sibship sizes increase, i.e. children in large sibships benefit less from parental resources. Here we investigate how direct measures of parental investment might mediate the relationship between family size and educational outcomes.

We contribute to this complicated literature by using rich detailed data on a nationally representative cohort of school children from England. Considering both the theoretical framework and the empirical evidence from the literature, we aim to address the following research questions:

1. Is sibship size associated with children's school attainment differentially by family SES?
2. Is direct parental investment able to mitigate the influence of siblings, and how does this differ across socioeconomic groups?

To answer these questions, we investigate the associations between sibship sizes and children's test scores in English and maths General Certificate of Secondary Education (GCSE) exams for different socioeconomic groups in England. We then introduce two measures of material and non-material parental investment to test if and how this mediates observed associations across socioeconomic groups.

## Methods

### Data

We use data from wave 1 of the Next Steps cohort (formerly the Longitudinal Study of Young People in England) collected in 2004, comprising 15,770 young people (74% of the target sample), aged 14 years (born 1989/90) who were enrolled in state and independent schools across England (University College London, 2018). We link these data to the National Pupil Database, which provides national school grades for all children in England (University College London, 2020). The surveys were conducted in face-to-face interviews where a battery of questions about the young person (YP) was asked of the ‘main parent’ (assigned as the parent who is most involved with the young person, unless there is only one parent). The primary sampling unit was schools (*n* = 652) with over-sampling of deprived schools and ethnic minorities, but is otherwise nationally representative. As such, we use appropriate methods to account for clustering.

### Sample selection

We excluded those for whom there were no data on the GCSE test scores (i.e. those who did not consent to sharing their data), and those with missing values for sibship size (our primary independent variable). Missingness on all other variables was imputed using multiple imputation methods (see below). We also removed children who were in institutional care at the time of interview, leaving a final linked sample of 14,257 young people.

### Variables

#### Test scores

The test scores are special licence access data from the National Pupil Database. We use the young person's English and maths scores attained in the GCSE mandatory exams taken at age 16 years (Key Stage 4). The test scores provided by the National Pupil Database are measured in grades ranging from ‘A’ (highest) to ‘G’ (lowest). We coded this as a scale from 1 to 7, with 7 representing the highest grade, ‘A’.

#### Sibship size

This is a variable from the question ‘How many [older/younger] brothers and sisters does YP have altogether?’ (Centre for Longitudinal Studies, 2004). Given that the focal child in our proposed analysis was 14 years old at the time of survey, it is unlikely that very many siblings had already left home, but even if they had they would probably have shared most of their childhood in the same household. It is also possible that more siblings may be born into the family after the study period but new babies in the household will be unlikely to be competing for the same resources that older children need. The phrasing of this question means that half- and step-siblings are included in the measure, and both male and female. We derived dummy variables for each sibship size: 0, 1, 2, 3, 4, 5, 6+.

#### Socioeconomic status

We performed a principal components analysis (PCA) to derive a measure of SES based on (a) the highest educational qualification of the main parent, (b) the household income band and (c) the occupational class of the main parent. A PCA is a data reduction method that produces one (or more) index variables (components) from a larger set of correlated, measured variables. It does this by estimating the weighted average of the larger set of variables in an optimal way. We then divided the SES component into deciles because we are most interested in how the ends of the distribution behave compared with the broad middle. With a sample size of around 1200 in each decile, we believe we can capture fine-grained information about within-group ecological variation.

#### Parental investment

The Next Steps study has collected a large number of variables for approximating parental involvement in the child's life and schooling. We performed one PCA for each set of variables given in Appendix A, and selected only the one component with the highest eigenvalue. The two types of parental investment that we use are:

1. parental involvement in child's schooling; and
2. parental aspirations for the child

#### Birth order

Birth order is highly correlated with sibship size, which is problematic for the research we propose. One way to get around this is to create an adjusted birth order measure as:
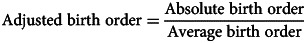
where average birth order is calculated as (sibship size +1)/2 which deflates the weight of absolute birth order (Booth & Kee, 2009). The deflated correlation between sibship size and birth order is thus substantially reduced; using census data from England and Wales, the Pearson's correlation coefficient dropped from 0.68 to 0.09 (Chan et al., 2019).

#### Controls

The controls were:

1. sex;
2. ethnicity (White, Asian (Indian, Pakistani, Bangladeshi), Black, and ‘other’ which includes ‘mixed’ and Chinese);
3. single-parent household;
4. birth order.

Birth order is highly correlated with sibship size which is problematic for the research we propose. One way to get around this is to create an adjusted birth order measure as:
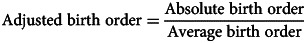
where average birth order is calculated as (sibship size +1)/2, which deflates the weight of absolute birth order (Booth & Kee, 2009). The deflated correlation between sibship size and birth order is thus substantially reduced; using census data from England and Wales, the Pearson's correlation coefficient dropped from 0.68 to 0.09 (Chan et al., 2019).

### Missing data

We performed multiple imputation methods to impute missing values on all of the dependent variables where there were missing values, except for the two outcome variables (English and maths GCSE scores) and our primary predictor variable: sibship size (see Table 1). Although there is no consensus about whether to impute missing values on the primary independent variable of interest, we were reluctant to impute values based on predictions made by the covariates in our models. Multiple imputation is the most appropriate way to account for missing data because it is an iterative process that can estimate more accurate standard errors than other known methods. The number of imputations necessary was determined using the two-stage calculation devised by Paul von Hippel (2016), by first performing a small number of pilot imputations and then, based on these results, calculating the optimal number of imputations with replicable standard error estimates.

**Table 1.** Missingness for all variables in the models

Variable	*n*	%
Maths^a^	1,484	10.4
English^a^	1,294	9.0
Ethnicity	273	1.9
Single-parent household	22	0.2
household income band	7,753	54.4
Highest qualification of main parent	428	3.0
Occupational status of main parent	360	2.5
Parental involvement	576	4.0
Parental aspirations	1,137	8.0

a Not imputed.

Total *N =* 14,257.

### Analysis

1. We provide summary statistics for all variables, stratified by sibship size (Table 2).
2. We derive principal components for:
  - SES based on education and occupation of main parent, and household income, which is divided into deciles;
  - two parental investment variables based on survey questions provided in Appendix A.
3. We then conduct multilevel ordinary least squares regression models to estimate the association between sibship size and GCSE grades for each SES decile. A random effect for school is included to account for non-independence of pupils clustered in schools, capturing unobserved heterogeneity within schools and, by extension, neighbourhoods.
4. We rerun the models, now including each of the parental investment variables, first in separate models and then altogether (Tables 3 and 4).

**Table 2.** Descriptive summaries of control variables by sibship size

*N* (%)								
	Only child	One sibling	Two siblings	Three siblings	Four siblings	Five siblings	Six+ siblings	Total
Male	544 (7.52)	2,648 (36.62)	2,047 (28.30)	1,098 (15.18)	439 (6.07)	224 (3.10)	232 (3.21)	7,232
Female	511 (7.27)	2,511 (35.74)	1,977 (28.14)	1,053 (14.99)	459 (6.53)	240 (3.42)	274 (3.90)	7,025
White	763 (7.99)	4,055 (42.44)	2,819 (29.51)	1,200 (12.56)	404 (4.23)	154 (1.61)	159 (1.66)	9,554
Asian	71 (2.93)	449 (18.52)	587 (24.22)	566 (23.35)	323 (13.33)	194 (8.00)	234 (9.65)	2,424
Other ethnicity	197 (9.82)	559 (27.87)	546 (27.22)	343 (17.10)	151 (7.53)	106 (5.28)	104 (5.18)	2,006
Single-parent household	393 (10.78)	1,231 (33.75)	952 (15.25)	556 (15.25)	248 (6.80)	119 (3.26)	148 (4.06)	3,647
Both parents in household	662 (6.25)	3,926 (37.08)	3,059 (28.89)	1,591 (15.03)	648 (6.12)	344 (3.25)	358 (3.38)	10,588

Note: ‘Other ethnicity’ includes ‘mixed’, Chinese, and ‘other’, collapsed together owing to small cell sizes in those categories.

**Table 3.** Results for multilevel linear regression models for GCSE maths grades

	Model 1			Model 2			Model 3			Model 4			Model 5			Model 6		
	*β*	LB	UB	*β*	LB	UB	*β*	LB	UB	*β*	LB	UB	*β*	LB	UB	*β*	LB	UB
Only child	—	—	—	—	—	—	—	—	—	—	—	—	—	—	—	—	—	—
One sibling	0.09	−0.01	0.19	0.01	−0.10	0.11	−0.03	−0.13	0. 08	−0.02	−0.12	0.08	0.03	−0.06	0.12	0.03	−0.06	0.12
Two siblings	−0.06	−0.17	0.04	−0.17**	−0.28	−0.64	−0.12*	−0.23	−0.02	−0.11*	−0.21	−0.01	−0.05	−0.15	0.05	−0.04	−0.14	0.05
Three siblings	−0.25***	−0.37	−0.14	−0.39***	−0.51	−0.27	−0.26***	−0.38	−0.14	−0.23***	−0.34	−0.11	−0.15**	−0.26	−0.04	−0.14*	−0.25	−0.03
Four siblings	−0.44***	−0.58	−0.30	−0.59***	−0.73	−0.44	−0.39***	−0.53	−0.25	−0.34***	−0.48	−0.20	−0.28***	−0.41	−0.15	−0.25***	−0.38	−0.12
Five siblings	−0.65***	−0.82	−0.48	−0.80***	−0.97	−0.62	−0.54***	−0.71	−0.37	−0.51***	−0.68	−0.34	−0.44***	−0.60	−0.28	−0.43***	−0.59	−0.27
Six+ siblings	−0.62***	−0.79	−0.45	−0.77***	−0.94	−0.60	−0.53***	−0.70	−0.36	−0.47***	−0.63	−0.30	−0.40***	−0.56	−0.24	−0.37***	−0.53	−0.22
Male				−0.06*	−0.11	−0.01	−0.06*	−0.11	−0.01	−0.03	−0.08	0.02	0.10***	0.06	0.15	0.11***	0.64	0.16
Single parent				−0.46***	−0.52	−0.40	−0.18***	−0.25	−0.12	−0.13***	−0.20	−0.74	−0.20***	−0.25	−0.14	−0.17***	−0.22	−0.11
White				—	—	—	—	—	—	—	—	—	—	—	—	—	—	—
Asian				0.26***	0.17	0.35	0.51***	0.43	0.60	0.48***	0.39	0.56	−0.04	−0.12	0.05	−0.03	−0.11	0.05
Mixed				0.02	−0.10	0.14	−0.01	−0.13	0.11	0.01	−0.11	0.13	−0.22***	−0.33	−0.11	−0.20***	−0.30	−0.09
Black				−0.09	−0.20	0.02	−0.18**	−0.29	−0.08	−0.23***	−0.33	−0.12	−0.61***	−0.71	−0.51	−0.61***	−0.71	−0.52
Other				0.36**	0.10	0.62	0.43**	0.17	0.69	0.39**	0.14	0.64	−0.02	−0.26	0.22	−0.02	−0.25	0.22
Adjusted birth order				−0.05*	−0.10	−0.01	−0.06**	−0.11	−0.02	−0.05*	−0.10	−0.01	−0.01	−0.05	0.04	0.01	−0.04	0.05
1 Lowest SES							—	—	—	—	—	—	—	—	—	—	—	—
2							−0.14*	0.02	0.27	0.15*	0.02	0.27	0.16*	0.04	0.28	0.16**	0.04	0.28
3							0.35***	0.22	0.47	0.35***	0.23	0.48	0.33***	0.21	0.44	0.33***	0.22	0.45
4							0.58***	0.45	0.71	0.59***	0.46	0.72	0.53***	0.41	0.65	0.53***	0.42	0.66
5							0.71***	0.59	0.84	0.74***	0.61	0.86	0.61***	0.49	0.73	0.63***	0.52	0.75
6							0.84***	0.71	0.97	0.87***	0.74	1.00	0.72***	0.60	0.84	0.75***	0.63	0.86
7							0.91***	0.78	1.04	0.94***	0.81	1.07	0.75***	0.62	0.87	0.77***	0.65	0.89
8							1.04***	0.91	1.18	1.07***	0.94	1.20	0.83***	0.71	0.96	0.86***	0.74	0.98
9							1.20***	1.07	1.33	1.24***	1.11	1.36	0.87***	0.75	0.99	0.91***	0.79	1.03
10 Highest SES							1.58***	1.44	1.71	1.62***	1.49	1.75	1.13***	0.99	1.25	1.18***	1.05	1.30
Involvement										0.19***	0.17	0.21				0.11***	0.10	0.13
Aspirations													0.76***	0.73	0.79	0.72***	0.69	0.75
Constant	—^a^	—	—	4.90***	4.75	5.05	4.06***	3.89	4.23	3.93***	3.76	4.09	3.95***	3.79	4.11	3.89***	3.73	4.04

*N* = 12,773 (in 652 schools).

LB and UB refer to lower and upper bounds of 95% confidence intervals.

Eighty-nine imputations, based on von Hippel (2016).

*** *p* < 0.001 ** *p* < 0.01 *p < 0.05.

a Constant not shown following UK data Service (UKDS) secure data stipulations.

**Table 4.** Results for multilevel linear regression models for GCSE English grades

	Model 1			Model 2			Model 3			Model 4			Model 5			Model 6		
	*β*	LB	UB	*β*	LB	UB	*β*	LB	UB	*β*	LB	UB	*β*	LB	UB	*β*	LB	UB
Only child	—	—	—	—	—	—	—	—	—	—	—	—	—	—	—	—	—	—
One sibling	0.07	−0.02	0.16	0.01	−0.09	0.10	−0.02	−0.10	0.07	−0.01	−0.10	0.08	0.04	−0.05	0.12	0.04	−0.04	0.12
Two siblings	−0.10*	−0.20	−0.01	−0.18***	−0.28	−0.09	−0.13**	−0.23	−0.04	−0.11*	−0.21	−0.02	−0.06	−0.15	0.02	−0.06	−0.14	0.03
Three siblings	−0.31***	−0.41	−0.21	−0.41***	−0.51	−0.30	−0.28***	−0.29	−0.18	−0.25***	−0.35	−0.15	−0.19***	−0.28	−0.09	−0.18***	−0.27	−0.08
Four siblings	−0.44***	−0.57	−0.32	−0.55***	−0.68	−0.42	−0.36***	−0.49	−0.24	−0.32***	−0.44	−0.20	−0.26***	−0.38	−0.15	−0.24***	−0.36	−0.13
Five siblings	−0.64***	−0.57	−0.32	−0.75***	−0.91	−0.60	−0.52***	−0.67	−0.37	−0.48***	−0.61	−0.33	−0.43***	−0.57	−0.29	−0.41***	−0.55	−0.27
Six+ siblings	−0.60***	−0.76	−0.45	−0.72***	−0.87	−0.57	−0.48***	−0.63	−0.34	−0.43***	−0.58	−0.29	−0.37***	−0.51	−0.24	−0.35***	−0.48	−0.21
Male				−0.50***	−0.54	−0.45	−0.50***	−0.55	−0.46	−0.47***	−0.52	−0.43	−0.36***	−0.40	−0.32	−0.35***	−0.39	−0.31
Single parent				−0.40***	−0.45	−0.34	−0.14***	−0.20	−0.09	−0.09**	−0.15	−0.04	−0.15***	−0.20	−0.10	−0.12***	−0.17	−0.07
White				—	—	—	—	—	—	—	—	—	—	—	—	—	—	—
Asian				0.19***	0.11	0.27	0.42***	0.35	0.50	0.39***	0.31	0.46	−0.07	−0.14	0.01	−0.06	−0.13	0.01
Mixed				0.13*	0.03	0.24	0.11*	0.01	0.21	0.13*	0.03	0.23	−0.08	−0.18	0.01	−0.06	−0.15	0.04
Black				0.07	−0.03	0.17	−0.02	−0.11	0.08	−0.6	−0.15	0.03	−0.39***	−0.48	−0.31	−0.40***	−0.48	−0.31
Other				0.17	−0.06	0.40	0.22*	0.01	0.45	0.20	−0.02	0.42	−0.19	−0.39	0.02	−0.17	−0.34	0.03
Adjusted birth order				−0.03	−0.07	0.01	−0.04*	−0.09	−0.01	−0.03	−0.07	0.02	0.01	−0.03	0.05	0.02	−0.02	0.06
1 Lowest SES							—	—	—	—	—	—	—	—	—	—	—	—
2							0.14*	0.03	0.25	0.15*	0.03	0.26	0.16**	0.05	0.26	0.16**	0.06	0.26
3							0.34***	0.23	0.45	0.34***	0.24	0.45	0.33***	0.22	0.43	0.33***	0.23	0.43
4							0.52***	0.41	0.64	0.53***	0.42	0.64	0.48***	0.37	0.58	0.49***	0.38	0.59
5							0.67***	0.55	0.78	0.69***	0.58	0.81	0.59***	0.48	0.70	0.61***	0.51	0.72
6							0.80***	0.68	0.91	0.82***	0.71	0.94	0.69***	0.59	0.80	0.72***	0.61	0.82
7							0.88*	0.76	0.99	0.91***	0.79	1.02	0.73***	0.62	0.84	0.76***	0.65	0.87
8							0.98	0.87	1.10	1.01***	0.89	1.12	0.78***	0.68	0.89	0.81***	0.70	0.91
9							1.17***	1.05	1.29	1.21***	1.10	1.33	0.88***	0.77	0.98	0.92***	0.81	1.03
10 Highest SES							1.47***	1.35	1.60	1.52***	1.40	1.64	1.07***	0.95	1.18	1.12***	1.01	1.24
Involvement										0.18***	0.17	0.20				0.11***	0.10	0.13
Aspirations													0.69***	0.66	0.71	0.64***	0.61	0.67
Constant	—^a^	—	—	5.32***	5.18	5.46	4.53***	4.38	4.68	4.40***	4.24	4.54	4.43***	4.29	4.57	4.35***	4.21	4.49

*N =* 12,963 (in 650 schools).

LB and UB refer to lower and upper bounds of 95% confidence intervals.

One-hundred and forty-three imputations, based on von Hippel (2016).

*** *p* < 0.001 ** *p* < 0.01 *p < 0.05.

a Constant not shown following UKDS secure data stipulations.

All analyses were performed using Stata v. 16. This study was preregistered on Open Science Framework (https://osf.io/74nr8/).

## Results

**Figure 1.**
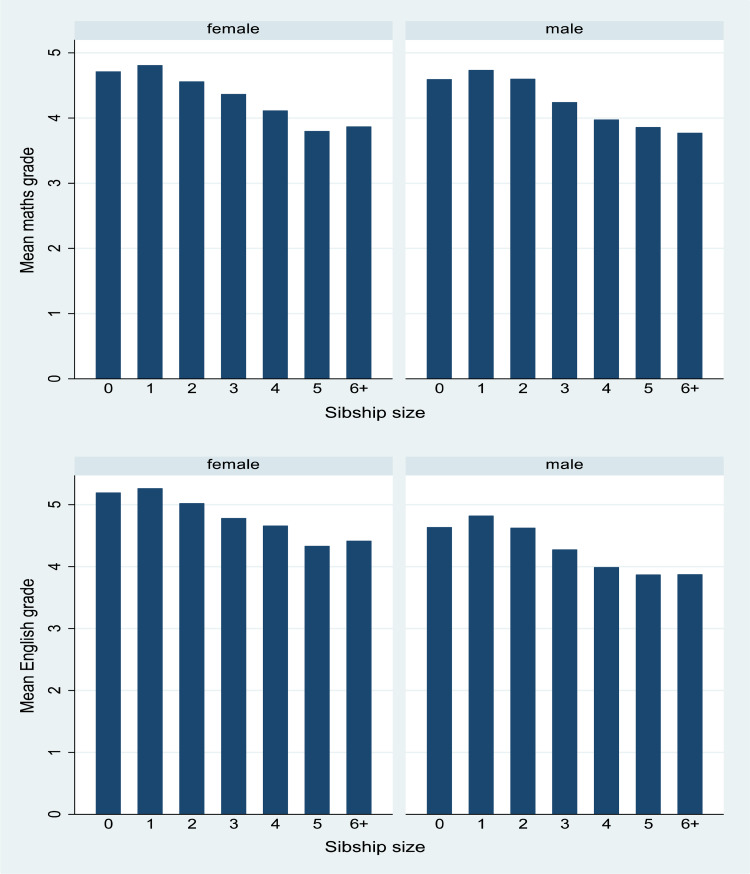
Mean GCSE grades by sibship size for males and females: maths (top) *n =* 12,773; English (below) *n =* 12,963.

**Figure 2.**
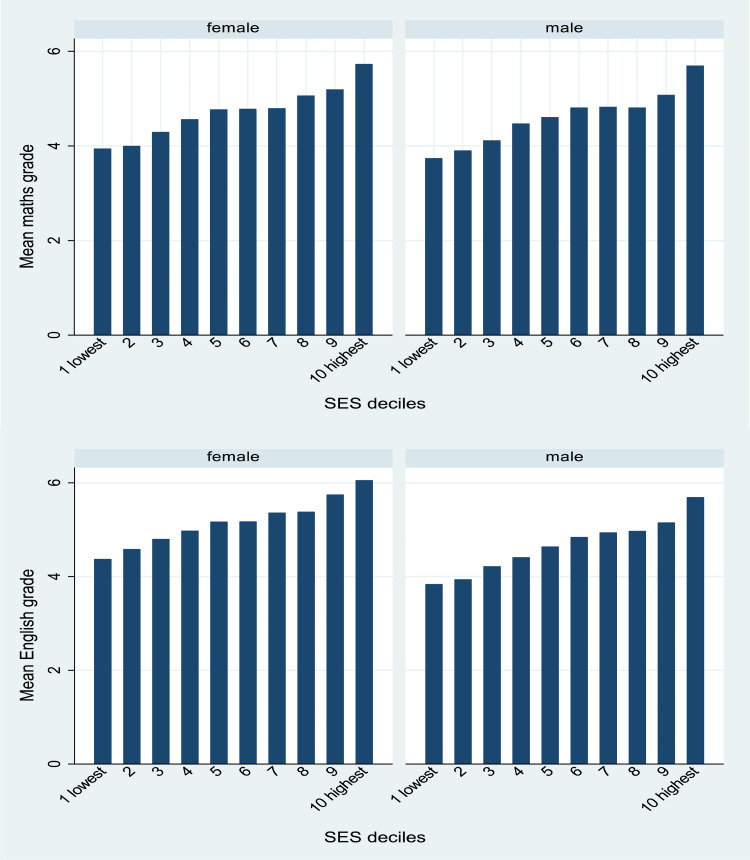
Mean GCSE grades by SES deciles for males and females: maths (top) *n =* 12,773; English (below) *n =* 12,963.

Summary statistics for categorical variables by sibship size are given in Table 2. Large families are more common among non-White groups. Figure 1 shows mean maths and English grades stratified by sibship size and Figure 2 shows maths and English grades, stratified by SES. We first examined these distributions for boys and girls separately to check if there were differences but besides girls performing slightly better than boys in English overall, there were no obvious differences and so we were satisfied to conduct our analyses with simply a control for sex. Note also, that the models for English (*n =* 12,963) have a slightly larger sample size than for maths (*n =* 12,773) as the students who opted out were not the same across the two subjects. The descriptive patterns suggest a negative, and fairly linear but not monotone, relationship between sibship sizes and grades, for both boys and girls, and for both English and maths. Only-children have slightly lower outcomes than children with one sibling. A positive pattern is observed for grades by SES.

## Maths

Table 3 shows results for six models with the outcome of GCSE maths grades. Model 1, the simplest model with only sibship size predicting GCSE maths grades, indicates that having more siblings is associated with poorer maths grades (in a more or less linear way). Compared with only-children, this is statistically significant for children with three siblings or more (i.e. there are four children in the family). On controlling for child gender, single-parent household, ethnicity and adjusted birth order (Model 2), the association is somewhat stronger (and is now significant for sibship sizes of three or more). Children with two or more siblings can expect to do worse in maths than their peers from families with one or two children. All of the other variables are also significantly associated with maths scores, with boys doing slightly worse than girls, and children from single-parent homes attaining on average almost half a grade lower than those from dual parent homes. Asian and ‘other’ ethnicity children do better in maths than other ethnic categories, and higher-birth-order children fare a little worse than early-borns.

Model 3 includes SES deciles. The general pattern by sibship size remains although the size of the differences is somewhat attenuated. Socioeconomic status itself is also associated with poorer maths grades in a generally linear fashion: children from the highest SES group score a grade around 1.6 higher than those from the lowest SES decile. Moving up each level of SES is associated with a further increase in expected grades. The effects of ethnicity are more pronounced once we control for SES.

In Models 4 and 5 we included variables for parental involvement and aspirations. Model 4, that includes a variable for parental involvement, does not significantly change the relationships observed in Model 3, although parental involvement itself is associated with higher maths grades. There is no longer a significant negative effect of being male, however. When parental aspiration is included instead (Model 5), the pattern by sibship size is somewhat attenuated again, but the overall pattern of lower scores among children with more siblings persists. Child sex now has the opposite direction compared with earlier models; males do better when controlling for parental aspirations. Model 6, which includes both parental investment variables, shows the same patterns in Model 5 and both parental investment variables remain statistically significant.

**Figure 3.**
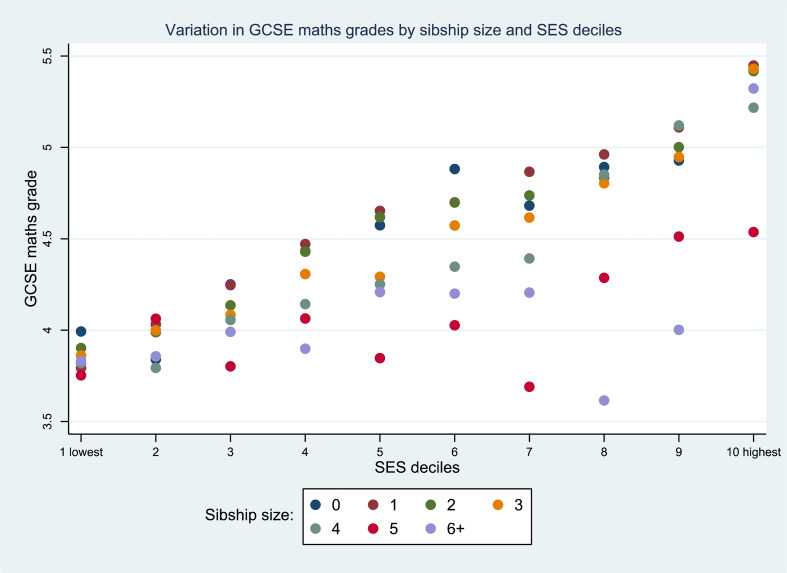
Predicted margins for interactions between SES and sibship size; maths (*n =* 12,773).

In a final model we included an interaction term between sibship size and SES deciles because we were interested in how the relationship between school grades and family size might be especially pronounced at the ends of the SES spectrum. In fact, we found no significant interactions. Figure 3 shows the predicted margins plot of the interactions. Note that the number of cases of children from families with four or more siblings is relatively small in the highest deciles. There is no clear pattern of interactions. By and large, the negative relationship between sibship size and maths grades is found across SES deciles.

## English

Model 1, the simplest model with only sibship size predicting GCSE English grades, as with maths, indicates that having more siblings is associated with poorer grades (more or less linearly). The negative association with family size is roughly similar in magnitude for English and maths. As with math scores, on controlling for child gender, single-parent household, ethnicity and birth order, the association with sibship size remains: children from three-child families or larger fare worse than peers from smaller sibships.

In Model 3, we included SES deciles, and like with maths, this somewhat attenuates the size of the differences but does not change the overall pattern: children with one or no siblings do better at English than all bigger family sizes. Again, SES is negatively associated with English grades in a monotonic way, and children from the highest SES score almost 1.5 grade points higher than those from the lowest decile. Boys consistently perform a little worse than girls on English.

Adding a variable for parental involvement (Model 4) does not change these associations but again, after including parental aspirations (Model 5) the small difference between the scores of children with two siblings and only-children is no longer significant. Model 6 includes both parental investment variables and, like with the maths model, this does not change the overall pattern, although it attenuates the size of the differences. Both parental involvement and aspirations are statistically significant, and their effect sizes are comparable with the models for maths.

**Figure 4.**
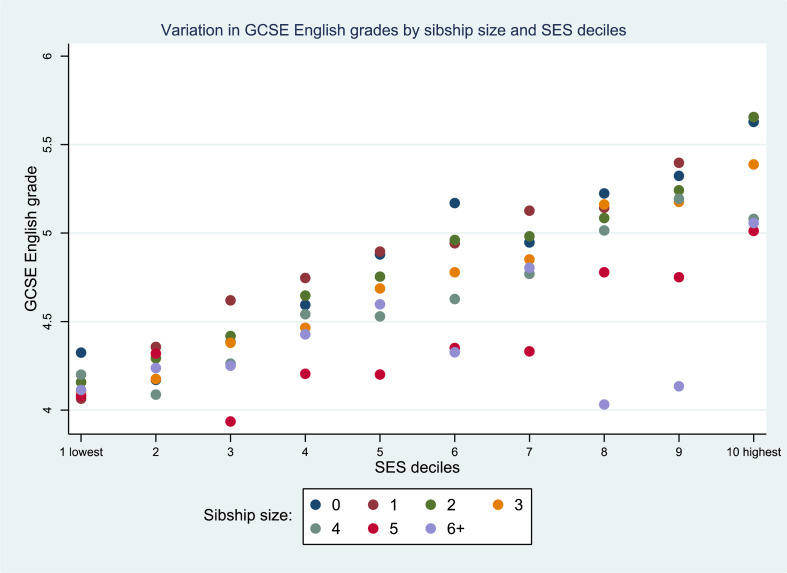
Predicted margins for interactions between SES and sibship size; English (*n =* 12,963).

As with maths, we found no significant interactions between SES and sibship size, and the margins plot (Figure 4) looks very similar to the maths model, although in general pupils perform slightly better in English than they do in maths.

## Robustness

To ensure that our findings were not influenced by high amounts of missing data imputed on household income, we re-ran all the models with a slightly different SES variable – a composite measure (principal component) of only the main parent's highest educational level and their occupation (i.e. omitting household income). The results from all the models do not change and the interaction plots are almost identical.

## Discussion

We set out to test if larger sibship sizes are negatively associated with school grades in the UK. Evolutionary theories of parental investment and economic models of resource dilution both led us to expect that children from large sibships would benefit less than those from small families. Our results here are largely in line with this prediction, with only small differences between results for English and those for maths. We thought too that we would find interesting differences at the upper and lower ends of the socioeconomic spectrum. We predicted that in the most wealthy and the least wealthy families sibship size would be less important than for those from the range in the middle because wealthier families can offset the costs of investment while poorer families might invest little no matter how many children there are. Perhaps our most striking finding is that there is no clear interaction between sibship size and SES and that even in the wealthier deciles, where on average children score better, those from larger families still score lower than their peers from smaller families, for both maths and English. We believe this finding to be methodologically robust and therefore substantively meaningful. One interpretation might be that even the wealthiest families are unable to compensate for the disadvantage that large family size might bring, at least in the UK. This might be surprising in a system where parents can invest in extra-curricular tutorials and other support, and raises questions about what the mechanisms for the association between family size and educational outcomes are. It may merit more research into birth-order effects, although we found little evidence for that here.

We found that having only one sibling as opposed to being the only child is not detrimental; the negative association only emerges at larger sibship sizes. This is in line with previous findings (Choi & Monden, 2017; Kalmijn & Werfhorst, 2016). It is important to bear in mind that there is (negative) selection into an only-child family, whereas the two-child family is the norm in low-fertility settings (Sobotka & Beaujouan, 2014). For instance, being in an only-child family might be due to parental relationship dissolution, but we cannot test this here. The difference between only-child and two-child families is probably driven more by selectivity of the only-child family, rather than by resource dilution.

### Parental investment

We were especially interested to test if direct measures of parental investment in children (here measured as parental involvement and aspirations) were able to offset the negative associations of large sibship sizes. Our models show that parental aspirations can compensate for having one extra child in both maths and English. This was not the case for parental involvement on its own but both of those variables exerted an independent, positive influence on school grades. The involvement variable captures information about the parent's interaction with the child's schooling, such as helping with homework and attending parent–teacher meetings. Aspirations is chiefly about the parent's desires for the child to progress to higher education. The impact of parental aspirations should be interpreted with caution as it is impossible to know the direction of the causal relationship. Do aspirational parents nurture their children's educational future, or do children who show academic promise nurture their parents’ aspirations?

Our findings are in line with findings from another British study where Lawson and Mace (2009) showed that parents of larger families invested less in their children, to the extent that ‘[e]ach additional sibling markedly reduces the amount of care that both mother and father give to each child’ (p. 177). As such, parental investment can only go so far and indeed our data suggest that in larger families (four children or more), neither involvement nor aspirations can mitigate the risk of poorer grades.

Neither of the parental investment variables we used attenuated the impact of SES on school grades, which is surprising and counter to the finding that parental interest in schooling significantly mitigated the risk that low SES had for having no qualifications in adulthood, also in the UK (Hango, 2007). Another study, using the same British data (1958 birth cohort) found that paternal involvement was positively associated with IQ scores at age 11 (Nettle, 2008). However he also showed that this association was stronger for fathers in higher-level occupations and attributes this to these fathers being more able to have an effect on their children's cognitive development than those with low socioeconomic capital. In other words skilled fathers may better know how to embody social capital in their children.

### Birth order

As outlined in the introduction, the evidence is mixed with regard to the importance of birth order vs. sibship size effects. Some studies have shown that birth order may be more important than sibship size (Barclay, 2015; Gibson & Sear, 2010; Härkönen, 2014; Kristensen & Bjerkedal, 2010) but we did not find evidence in support for this here. The measure of birth order used here has a statistically significant but substantively small effect on maths grades. Moreover, it becomes non-significant after entering the parental involvement variables. Birth order does not attenuate the associations with sibship size as one would expect if it was very important. We have used an adjusted measure of birth order to reduce the strong correlation between birth order and sibship size. This has been shown to be an effective way to model both variables simultaneously (Booth & Kee, 2009; Chan et al., 2019). The other studies mentioned above handle this problem in different ways so it is difficult to properly compare across studies. Furthermore, in an evolutionary ecological framework we might not expect all studies to agree; it is likely that the relative importance of both sibship size and birth order depends on the context.

Along these lines, we did expect to see interesting differences at the ends of the socioeconomic spectrum but this was not the case. Interactions between SES and sibship size were not statistically significant and the plots show a linear relationship across the SES deciles – children from small families do better in all SES deciles. Overall, our results show that, while low SES is associated with lower educational outcomes in England, children from larger sibships have lower outcomes than their counterparts from smaller families across the board, regardless of birth order.

It was something of a limitation that the question regarding siblings captured all siblings including half- and step-siblings, so we could not eliminate the possibility of parental discrimination towards some siblings based on genetic relationships. Future work that is able to discern differing effects of biologically related siblings might reveal more nuanced associations between sibling sizes and educational attainment. Similarly, studies that focus on same-sex sibship sizes may also uncover interesting patterns that we could not find here.

## Research transparency and reproducibility

Original registered report can be found at https://osf.io/74nr8/. National Pupil Database data are held by the UK Data Service under special licence which can be applied for from them.

## Appendix A: Variables used in the principal components analyses for parental investment

**Table:**

Q. code	Attitude to young person's school and involvement in education	Possible responses	Intended recode
Kidskol	How would you rate the overall quality of YP's school?	1 (very good) to 5 (very bad)	
Qualprg	How satisfied have you been with YP's school progress in general?	1 (very satisfied) to 4 (very dissatisfied)	
Qualdec	How satisfied have you been with the subjects YP has on offer at school?	1 (very satisfied) to 4 (very dissatisfied)	
Qualint	How satisfied have you been with how much interest the teachers show in YP?	1 (very satisfied) to 4 (very dissatisfied)	
Qualdis	How satisfied have you been with discipline at YP's school?	1 (very satisfied) to 4 (very dissatisfied)	
Qualrel	How satisfied have you been about how well YP gets on with other young people at school?	1 (very satisfied) to 4 (very dissatisfied)	
ParEve	Have you gone to any parent's evenings or similar events at YP's school?	Yes – respondent and partner/husband/wife have gone Yes – respondent went alone Yes – respondent's partner/husband/wife went alone No – Nobody has been	= yes (1–3) = No (4)
TmeetF	Apart from parents’ evenings, in the last 12 months, have you had any specially arranged meetings with teachers about how YP is getting on at school?	Yes No	
Tspeak	Apart from any parents’ evenings, how often do you talk to YP's teachers about how YP is getting on at school?	At least once a week Every two or three weeks At least once a term Less often than once a term Never?	
Schlif	How involved do you personally feel in YP's school life?	1 (very involved) to 4 (not at all involved)	
RepRed1	When you get YP's school reports, do you ever talk about them with YP?	Yes No Only if it's bad	
Teaclea	YP's school gives me clear information on how YP is getting on	1 (agree strongly) to 4 (disagree strongly)	
Teainv	YP's school makes it easy for me to get involved in YP's education	1 (agree strongly) to 4 (disagree strongly)	
Extrtu1	In the last 12 months have you or another member of your family paid for YP to have private classes or lessons in subjects that they also do at school?	Yes No	
Extrtu4	In the last 12 months have you, or another member of the family paid for any other classes or lessons for YP?	Yes No	
	*Parental expectations and aspirations*		
Patt1	Nowadays you need qualifications in order to get a job worth having?	1 (agree strongly) to 4 (disagree strongly)	
Patt2	Leaving school at 16 limits young people's career opportunities later in life?	1 (agree strongly) to 4 (disagree strongly)	
Patt3	I want YP to have a better education that I did	1 (agree strongly) to 4 (disagree strongly)	
Parasp2	What would you like YP to do when YP reaches 16 and can leave school?	Continue in full-time education Start learning a trade/Get a place on a training course Start an apprenticeship Get a full-time paid job (either as an employee or self-employed) Something else?	Combine 2 and 3, recode 5 into 1–4 if applicable keep as ‘other’ if gap year, etc.
Heposs	How likely is it that YP will go on to university to do a degree at some time in the future?	1 (very likely) to 4 (not likely at all)	

Scales also have a ‘can't say’ or ‘don't know’ option.

YP, young person.
